# Supplementary material for: The Systems Biology Research Tool: evolvable open-source software
Source: BMC Syst Biol. 2008 Jun 29;2:55. doi: 10.1186/1752-0509-2-55 (PMC2446383; doi:10.1186/1752-0509-2-55)
Supplement: Additional file 1 — SBRT Archive. An archive of the current version of the Systems Biology Research Tool. [file 1752-0509-2-55-S1.zip › sbrt-1.4.0/doc/developers_guide/api/sbrt/kernel/fba/comp/class-use/CycleComputer.html]

Uses of Interface sbrt.kernel.fba.comp.CycleComputer


|  |  |  |
| --- | --- | --- |
| **Overview** \| **Package** \| **Class** \| **Use** \| **Tree** \| **Deprecated** \| **Index** \| **Help** | |  |
| PREV   NEXT | **FRAMES**    **NO FRAMES**     **All Classes** |  |


---


## **Uses of Interface sbrt.kernel.fba.comp.CycleComputer**

| Packages that use CycleComputer | |
| --- | --- |
| **sbrt.kernel.fba.comp** | Provides classes and interfaces for performing Flux Balance Analysis computations. |
| **sbrt.kernel.fba.proc** | Provides classes and interfaces for executing Flux Balance Analysis processes. |

Uses of CycleComputer in sbrt.kernel.fba.comp

| Classes in sbrt.kernel.fba.comp that implement CycleComputer | |
| --- | --- |
| `class` | `SlpCycleComputer`             This class is used to identify the steady state cycles (Type III extreme pathways) in a fluxome. |

Uses of CycleComputer in sbrt.kernel.fba.proc

Constructors in sbrt.kernel.fba.proc with parameters of type CycleComputer

`CycleIdProcess(IrrevRxnFluxome fluxome,
CycleComputer computer)`
  
          Constructs a new steady state cycle identification process.

---


|  |  |  |
| --- | --- | --- |
| **Overview** \| **Package** \| **Class** \| **Use** \| **Tree** \| **Deprecated** \| **Index** \| **Help** | |  |
| PREV   NEXT | **FRAMES**    **NO FRAMES**     **All Classes** |  |


---
